# Autonomy-related Parenting Profiles and their Effects on Adolescents’ Academic and Psychological Development: A Longitudinal Person-oriented Analysis

**DOI:** 10.1007/s10964-021-01538-5

**Published:** 2021-11-22

**Authors:** Ziwen Teuber, Xin Tang, Lena Sielemann, Nantje Otterpohl, Elke Wild

**Affiliations:** 1 Department of Psychology, Bielefeld University, Bielefeld, Germany; 2 School of Psychology, Central China Normal University, Wuhan, China; 3 Faculty of Educational Sciences, University of Helsinki, Helsinki, Finland; 4 Department of Psychology, Justus Liebig University Giessen, Giessen, Germany

**Keywords:** Parental conditional regard, Self-determination, Multiple informants, Latent profile and latent transition analyses, Psychopathology

## Abstract

The important role of parenting is widely acknowledged, but as most studies have understood and examined it as a stable attribute (e.g., parenting style), the stability of and changes in parenting are less well understood. Using longitudinal person-oriented approaches (i.e., latent profile analyses and latent transition analyses), this study aimed to examine the stability of and changes in autonomy-related parenting profiles and their effects on adolescents’ academic and psychological development. Four autonomy-related dimensions (i.e., autonomy support, warmth, psychological control, conditional regard) were chosen to identify parenting profiles on the basis of Self-Determination Theory. Using five-year longitudinal data from 789 German secondary school students (50.06% female, *M*_age_ at T1 = 10.82 years, age span = 10–17), four autonomy-related parenting profiles were found: *Supportive* (~17%), *Controlling* (~31%), *Unsupportive-Uncontrolling* (~17%), and *Limited Supportive* (~35%). The results suggest that the *Supportive* profile contributes to adolescents’ positive academic and psychological development, whereas the *Controlling* profile, which thwarts autonomy development, exacerbates the development of psychopathology, and impairs academic achievement. More importantly, the *Limited Supportive* profile is as maladaptive as the *Unsupportive-Uncontrolling* profile. Regarding parenting profiles’ stability and changes, the results showed that about half of each profile stayed in the same group. Overall, it could be observed that parents became more supportive and less controlling over time. However, the findings also indicate that parenting profiles are less stable than expected and can still change during early-to-mid adolescence.

## Introduction

Adolescence is generally acknowledged as a dynamic and critical stage of human development in which individuals rapidly develop capacities for independence, seek to become more autonomous from their parents, and make decisions that can frame their developmental pathways or trajectories (R. M. Ryan et al., 2006; Shlafer et al., 2014). It is also challenging for parents to teach adolescents fundamental values, to guide their regulations, and to support the development of their autonomy (Joussemet et al., 2008; R. M. Ryan et al., 2006). This study focused on the stability of and the changes in autonomy-related parenting profiles and their effects on adolescents’ academic and psychological development in the period from early adolescence to mid-adolescence.

Although various frameworks of parenting practices have been examined (Bornstein, 2015), this study investigated them from the perspective of Self-Determination Theory (Deci & Ryan, 2000). Self-Determination Theory postulates that human behaviors are driven by three universal and innate psychological needs, namely autonomy, competence, and relatedness. Autonomy––the sense of psychological liberty and freedom––weaves throughout the broad framework of Self-Determination Theory (Vansteenkiste et al., 2010) and plays an important role in young people’s internalization of societal norms and rules, the development of motivational orientations, and self-regulation (R. M. Ryan et al., 2006). From the perspective of Self-Determination Theory, adolescents’ socialization contexts can be categorized as autonomy-supportive contexts and controlling contexts (see also Assor et al., 2004). Inspired by this theory, autonomy-supportive parenting, characterized by acknowledging one’s child’s view and encouraging self-initiated activities, has proven to support students’ academic and psychological adjustment (for an overview, see Vansteenkiste & Ryan, 2013). In contrast, controlling parenting, which counteracts young people’s autonomy through, for example, psychological control and conditional regard, can significantly increase adolescents’ risks of developing psychopathology and impair their academic achievements (e.g., Assor et al., 2014; Otterpohl et al., 2019).

Instead of studying parenting as having discrete dimensions, this study used a person-oriented approach and aimed to identify the combinations of parenting dimensions as an undivided whole (Bergman & Trost, 2006; for a classic line of research, see Baumrind, 1995). More importantly, it further aimed to expand our knowledge in this area by examining autonomy-related parenting profiles’ stability and changes and their longitudinal effects on adolescents’ academic achievements, prosocial behavior, and psychopathology from early adolescence (i.e., 10–13 years, also preadolescence) to mid-adolescence (i.e., 14–17 years). To pursue these objectives, five-year longitudinal data were used within a large-scale German project that included both adolescents’ self-reports and parent reports. Latent profile analyses (LPA) and latent transition analyses (LTA) were employed to examine parenting profiles on the basis of dimensions of Self-Determination Theory.

### Parenting Dimensions from a Self-determination Perspective and their Effects

To date, the parenting literature has been dominated by the two classic parenting dimensions, namely parental responsiveness and parental demandingness (Maccoby & Martin, 1983). Responsiveness––also warmth––refers to being accepting, sensitive to children’s needs, and emotionally warm, whereas demandingness––also behavioral control––is defined as parenting practices in which parents communicate clear expectations in terms of appropriate behaviors and use rules, instructions, and restrictions to regulate and monitor their children’s behavior (Barber et al., 2005; Baumrind, 1995).

Self-Determination Theory is another theory that has been used to examine the role of parenting in adolescent development. According to this theory, critical parenting practices should support the need for autonomy (i.e., feeling psychological liberty and freedom of internal will), for competence (i.e., feeling able to affect one’s environment), and for relatedness (i.e., feeling bonded and cared for; Deci & Ryan 2000; Vansteenkiste et al., 2020). Although many parenting practices fall into these categories (e.g., *provision of structure*; Griffith and Grolnick, 2014), the most important practices are autonomy-related supportive practices as they are a prerequisite for the unfolding of positive effects of other supportive parenting practices (Deci & Ryan, 2000; Griffith & Grolnick, 2014). This study investigated four parenting practices––autonomy support, warmth, psychological control, and conditional regard––as they are the most relevant practices that link to the needs for autonomy. Although autonomy-related practices are assumed to be critical for children’s and adolescents’ development, a comprehensive examination of them is still lacking. The present study thus aimed to fill this gap with the following four dimensions.

Parental autonomy support refers to parents’ active support of their children’s capability to be autonomous and self-initiating by acknowledging their children’s perspectives, allowing and encouraging them to experiment, giving them opportunities to make choices, and providing explanatory rationales for specific expectations (Deci & Ryan, 2012). Meta-analytical (Vasquez et al., 2016) and cross-cultural (Vansteenkiste et al., 2005; Zhang et al., 2017) evidence has shown the positive effect of parental autonomy support on adolescents’ self-regulation, adaptive psychosocial functioning, and academic success. Like Baumrind’s theory, Self-Determination Theory vales *parental warmth* (a core aspect of involvement; for detail, see Grolnick, 2009). Empirical studies have also found that parental warmth is closely related to autonomy support (for detail, see R. M. Ryan et al., 2006). Self-Determination Theory regards parental warmth as integral to autonomy support. That is, adolescents feel warmly connected to their parents only to the extent that their real selves are accepted by their parents. In contrast, the quality of relatedness suffers if autonomy is perceived as absent (R. M. Ryan et al., 2006). By showing care, support, and compassion, parents can support their children’s needs for relatedness (Grolnick, 2009). Past research has shown parental warmth to have a positive effect on adolescent academic and psychological development as well as on prosocial behaviors (see meta-analysis, Pinquart, 2017).

The opposite of parental autonomy support is *parental psychological control*, which frustrates adolescents’ needs for autonomy (Joussemet et al., 2008; R. M. Ryan et al., 2006). Psychological control is defined as “parental control that intrudes on the child’s psychological world” (Joussemet et al., 2008, p. 195). Psychological controlling practices include withdrawing love, inducing guilt, shaming, and invalidating the child’s perspective (Barber et al., 2005). Previous studies have consistently shown that parental psychological control is related to lower academic achievement, more externalizing and internalizing symptoms, and fewer prosocial behaviors (e.g., Pinquart, 2017; Wong et al., 2021). Lastly, *parental conditional regard* (PCR) is the practice through which parents show love and appreciation when children fulfill their parental expectations (Assor et al., 2014). PCR can be divided into conditional negative regard (PCNR) and conditional positive regard (PCPR). In the former, parents withdraw attention and affection when the child fails to comply with their expectations, whereas in the latter, more attention and affection is given when the child acts as expected (Roth et al., 2009). Hence, parents who use PCR strategies engage in a controlling manner in their children’s development by providing relatedness at the expense of autonomy (Vansteenkiste et al., 2010). PCR and psychological control (e.g., love withdrawal) are closely related but also distinct from each other. First, psychological control is a general parenting technique, whereas PCR is domain specific (e.g., academic, affective, and behavioral domains). Second, psychological control contains components of blame that the child cannot change or influence through behavior, whereas PCR refers to showing esteem and attention depending upon the child’s behavior (Assor et al., 2014; Roth et al., 2009). Previous studies have highlighted how PCR has a wide range of detrimental effects on child and adolescent development. Both forms of PCR can cause introjected internalization of parents’ expectations, increase the child’s internal stress, diminish well-being (for an overview, see Assor et al., 2014), and increase the risk of self-regulation failures (e.g., Curran et al., 2017) and the development of internalizing and externalizing problems such as aggressive behaviors, negative emotions, anxiety, and depression (e.g., Otterpohl et al., 2019).

### Person-oriented Parenting Approaches

Although parenting dimensions contribute to a vast knowledge on the role of parenting in adolescent development (Bornstein, 2015), the combinations of various parenting dimensions (e.g., parenting styles/profiles) offer a deeper understanding of good parenting (Bornstein, 2015; Darling & Steinberg, 1993; Steinberg et al., 1992). One classic example is the four parenting styles based on responsiveness and demandingness (Maccoby & Martin, 1983): authoritative (high responsiveness and high demandingness), authoritarian (low responsiveness and high demandingness), permissive (high responsiveness and low demandingness), and neglectful (low responsiveness and low demandingness). However, this framework has been criticized for not addressing the important aspect of granting autonomy (e.g., Steinberg et al., 1992). Moreover, the method that creates these parenting styles/profiles is either a scale-mean method (i.e., plus or minus one *SD* of the mean as high and low levels of a dimension) or a median-split method (i.e., above and below the median as high and low levels of a dimension). These methods are arbitrary in defining cut-off points and are largely inappropriate when multiple dimensions (more than three) are used (Morin & Litalien, 2019). This study adopted a mixture of modeling-backed and person-oriented approaches that can reflect the natural configuration of the combinations of dimensions (Bergman & Trost, 2006). A further advantage of this approach is that its extension to latent transition analysis enables the estimation of the stability of and changes in group membership in profiles (Lanza et al., 2003; Morin & Litalien, 2019).

To date, comparatively fewer studies have examined parenting profiles based on dimensions of Self-Determination Theory, particularly autonomy-related parenting practices. Furthermore, the literature has not attempted to include PCR in the testing of parenting profiles. This study focused on the central idea of Self-Determination Theory, namely an autonomy-supportive vs. a controlling socialization climate at home, by incorporating key parenting dimensions (i.e., autonomy support, warmth, and psychological control) and conditional regard. Thus, unlike previous studies that have examined many parenting dimensions, this study sought to identify parenting profiles associated with adolescents’ needs for autonomy. According to Self-Determination Theory, parental autonomy support facilitates children’s experience of autonomy, whereas parental psychological control undermines children’s internal will and thus frustrates their needs for autonomy. Various researchers have regarded autonomy support and psychological control as two sides of the same coin (e.g., Joussemet et al., 2008; Yotyodying et al., 2020). Therefore, finding parents who are autonomy supportive and psychologically controlling is not very likely. However, from our point of view, low control does not equal autonomy support. The present study hypothesized a supportive parenting profile, characterized by high scores in both supportive dimensions (i.e., autonomy support and warmth) and low scores in both controlling dimensions (i.e., psychological control and conditional regard). Conversely, a highly controlling profile was also expected, characterized by low scores on the supportive dimensions and high scores on the controlling dimensions. Further, it was expected to find a profile in which parents are neither supportive nor controlling. PCR refers to parents’ domain-specific controlling parenting strategies. Following Assor et al. (2014), parents can be autonomy supportive in general and use these strategies in specific domains. Hence, it is reasonable to assume that some parents are generally supportive and regularly use conditional regard strategies.

### Parenting Profiles and Adolescent Development

Adolescence is a critical time in the development of academic and psychological functioning. The etiology of the development of academic and psychological maladjustment is multifactorial. Apart from biological, genetic, and situational predispositions, needs-thwarting parenting is seen as the most significant risk factor from the Self-Determination Theory perspective (W. S. Ryan & Ryan, 2019). To understand the role of autonomy-related parenting profiles in adolescent development, this study evaluated multiple domains of adolescent outcomes. These included academic achievement, internalizing and externalizing problems, and prosocial behavior. In the present study, internalizing problems refer to emotional problems, social withdrawal, and associated problems integrating into peer groups. Externalizing problems in turn include hyperactivity, distractions, and delinquent and aggressive behaviors (A. Goodman & Goodman, 2009). In contrast, prosocial behavior is a positive outcome linked to greater academic and social adjustment, and this promoting effect persists into adulthood (Brook et al., 2013). Furthermore, adolescents and their parents often provide divergent assessments of adolescents’ psychological outcomes (Lohaus et al., 2020), and this leads to difficulties in interpretation. In addition to adolescents’ self-reports, the present study also took parent reports of adolescents’ internalizing and externalizing problems and prosocial behavior into consideration.

As previously described, the positive effects of parental autonomy support and warmth, and the detrimental effects of psychological control and conditional regard on adolescents’ academic and psychological adjustment have been well documented. Attempts have been made to examine the effects of different parenting dimension constellations on adolescent outcomes. Several studies have incorporated some autonomy-related parenting dimensions. In a two-wave Portuguese study (Pereira et al., 2009), parenting profiles were created on the basis of parental warmth, rejection (a form of psychological control), and overprotection (low autonomy granting). Four parenting profiles were found and labeled *Low Support*, *Supportive-Controller*, *Rejecting-Controller*, and *Supportive*. Of these, the *Rejecting-Controller* profile (i.e., low warmth, high rejection, and high overprotection) was the theoretically most maladaptive profile and was related to the highest level of children’s behavioral problems, whereas the *Supportive* profile was the most adaptive profile associated with the lowest level of behavioral problems. A more recent study (Shen et al., 2020) focused on the effects of parenting profiles on internalizing problems among Chinese primary school children. The same parenting dimensions were used as those in the Portuguese study (Pereira et al., 2009), and similar profiles were found. The results further showed that the most maladaptive profile was associated with the highest risk of children’s emotional maladjustment. Some longitudinal studies (e.g., Kim et al., 2013; Zhang et al., 2017), which integrated autonomy support or psychological control into the classic two-dimension typology (Baumrind, 1995; Maccoby & Martin, 1983), have consistently shown that autonomy-granting belongs to adaptive parenting profiles and buffers adolescents’ development of psychopathology, whereas psychological control belongs to maladaptive profiles and increases the risk of psychopathology.

Based on these findings, it was expected that the supportive parenting profile would be linked to the lowest scores in adolescent internalizing and externalizing problems, the highest school performance, and the most prosocial behaviors, whereas inverse relationships were expected in the controlling profile. Being neither supportive nor controlling was expected to detrimentally affect adolescents’ development because their needs are not supported. If parents were generally supportive but showed conditional regard in specific domains, negative influences on adolescent outcomes were expected.

When examining the effects of autonomy-related parenting profiles on adolescent outcomes, multiple time-invariant confounders should be taken into consideration. This study was conducted in Germany, where the education system is characterized by high levels of aggregation and social inequality (OECD, 2019). For example, girls and students from high socioeconomic families are overrepresented in the highest track secondary school, whereas boys, students with a migrant background, and students from low socioeconomic families are overrepresented in the lowest track secondary school (Kessels et al., 2014). Moreover, socioeconomic status is associated with adolescents’ mental health and psychological adjustment (Klipker et al., 2018). Parenting styles/profiles vary as a function of social stratum (for an overview, see Hoff & Laursen, 2019) and can be additionally influenced by adolescents’ gender (for detail, see Bornstein, 2013). It has been demonstrated that parents of low socioeconomic status are more controlling in their parenting style (e.g., Benner et al., 2016), and that boys are more likely to experience controlling parenting than girls (e.g., Bornstein, 2013). Therefore, the present study included adolescents’ gender, school type, migration background, and socioeconomic status as covariates.

### Parenting Profiles’ Stability and Changes

In addition to the limitations described above, another drawback of the traditional parenting profile studies is that they assume that parenting styles/profiles are largely stable, or put little effort into examining changes in these profiles (e.g., Hoeve et al., 2008; Pereira et al., 2009). Parenting styles/profiles can indeed change, particularly in a period that witnesses drastic changes, such as adolescence (Kuczynski & Parkin, 2007; Sameroff, 2010). In a study with a sample of 2173 Chinese adolescent students, Zhang et al. (2017) identified four parenting profiles (authoritative, authoritarian, average-level undifferentiated, and strict-affectionate), based on six dimensions: warmth, inductive reasoning, encouragement of independence, encouragement of achievement, supervision, and harshness. From childhood to early adolescence, about one-third of authoritative, strict-affectionate, and average-level undifferentiated mothers changed parenting profiles. The profile stability of authoritarian parenting was 50–60%. The authors speculated that authoritarian parents shifted to other profiles due to the modification of their parenting behaviors. In their study of 444 Chinese American parent-adolescent dyads over eight years, Kim et al. (2013) found four parenting profiles (supportive, tiger, easygoing, and harsh) based on eight parenting dimensions (warmth, monitoring, democratic parenting, inductive reasoning, hostility, psychological control, shaming, and punitive parenting). The latent profiles were cross-sectionally examined and compared on the basis of the number of profiles and mean patterns. Their results showed that from early to late adolescence, the proportion of tiger mothers tended to decrease, whereas the proportion of tiger fathers tended to increase. These findings suggest that parents adapt their parenting to a more autonomy-supportive manner in response to their children’s increasing needs for autonomy.

To date, however, longitudinal studies of parenting profiles that highlight autonomy-related practices based on the Self-Determination Theory are still lacking. Consequently, the stability of and change in these profiles are far from well understood. On the other hand, research on the stability of and change in parenting profiles could greatly contribute to the understanding of parenting dynamics and their impact on adolescents’ academic and psychological development.

## Current Study

Despite the importance of supporting adolescents’ needs for autonomy, less is known about autonomy-related parenting profiles and their impact on adolescent development. Moreover, the stabilities of and changes in parenting profiles have rarely been addressed in previous research. Employing a longitudinal person-oriented approach, this study pursued three research objectives.

The first research objective of this study was to explore the number and characteristics of autonomy-related parenting profiles. Some parents were expected to be supportive by granting autonomy, showing warmth, and using minimal psychological control and conditional regard. In contrast, some parents were expected to be controlling by providing little autonomy and warmth and using psychological control and conditional regard. It was further hypothesized that some parents would be neither supportive nor controlling. Finally, some parents were expected to be generally supportive and regularly use conditional regard strategies.

The second research objective of this study was to estimate the stability of and changes in these profiles across a relatively long period of time (i.e., from early adolescence to mid-adolescence). It was assumed that the parenting profiles of some parents may stay relatively stable over time, but that some changes would also be observed. Moreover, it was hypothesized that more parents would become autonomy-supportive and less controlling over time.

The third and final research objective of this study was to examine the longitudinal effects of autonomy-related parenting profiles on adolescents’ academic and psychological outcomes. If the four hypothesized autonomy-related parenting profiles would be found, it was expected that adolescents who experienced supportive parenting would show the most positive outcomes, whereas adolescents who experienced controlling parenting would show the most negative outcomes. Negative adolescent outcomes were also expected to be found among adolescents whose parents were neither supportive nor controlling, and among those whose parents were generally supportive but used conditional regard.

## Method

### Participants and Procedure

The data were collected from 29 secondary schools in the state of North-Rhine Westphalia, Germany during a five-year longitudinal project (from the fifth grade to the ninth grade, four measurement points, with a 1–1–2-year interval, T1 in spring 2010, T2 in spring 2011, T3 in spring 2012, and T4 in spring 2014) entitled “Families’ Support in the Acquisition of Discourse- and Text Competence in Secondary School” (in German: *Die Rolle familialer Unterstützung beim Erwerb von Diskurs- und Schreibfähigkeiten in der Sekundarstufe 1*). Participation in the project was voluntary and signed informed consent forms were collected from both the adolescents and their parents. The participants were able to withdraw their participation at any time without any consequences. This project received the approval of the ethics review committee of Bielefeld University. The adolescent questionnaires were administered by trained instructors (i.e., student assistants) during school hours and took about an hour to complete. After the testing session at school, the adolescents took home an envelope containing parental questionnaires and written instructions for their parents. The parents were asked to return their completed questionnaires by post to Bielefeld University. After each measurement occasion, every parent–adolescent dyad received a voucher worth 15 euros for their participation.

In total, 884 students (mean age at T1 = 10.82 years, *SD* = 0.59, age span from T1 to T4 = 10–17) participated in all three waves. After removing the students who had not filled out the scales of parenting practices, 789 students (50.06% girls) remained in the analysis. Of these, 205 students attended the lowest track secondary school (*Hauptschule*, vocational track, grades 5–10), whereas 584 students attended the highest track secondary school (*Gymnasium*, academic track, grade 5–13). Furthermore, 245 participants had a migrant background (i.e., the student or at least one of their parents was not born in Germany). Appendix 1 presents detailed information on the original sample and the sample in this study.

The present study used parental reports on adolescents’ behaviors (assessed at T3 and T4). At T3, 579 parent reports (completed by 427 mothers, 60 fathers, 85 mother and father together, 1 someone else, and 6 no response) were included, whereas at T4 this figure was 533 (completed by 411 mothers, 60 fathers, 59 both parents, and 3 no response). At T3, the parent participants consisted of 127 parents of *Hauptschule* students and 452 parents of *Gymnasium* students. At T4, of the parent participants, 108 were parents of *Hauptschule* students and 425 parents of *Gymnasium* students. The proportion of parents who had a migrant background was 23.49% at T3 and 25.33% at T4.

Each adolescent–parent dyad generated an individual 13-digit code (combinations of numbers and letters) based on personal facts (e.g., the second letter of the given name of the child). This code was used for merging parent and adolescent data from different measurement time.

### Measures

Academic achievement, internalizing problems, externalizing problems, and prosocial behavior were modeled as manifest variables (i.e., scale mean; measured at T3 and T4). The four parenting dimensions had six facets: autonomy support (with two facets: parental acknowledgment of child’s perspective and encouragement of child-initiated activities), warmth, psychological control, and conditional regard (with two facets: PCNR and PCPR). These six facets were modeled as latent variables with multiple indicators (measured at T1, T3, and T4). McDonald’s omega (McDonald, 1999) is reported as a measure of internal consistency because it reflects the proportion of variance in the scale scores that is explained by a general latent factor (Zinbarg et al., 2006).

#### General parenting dimensions

The German Parental Behavior Scale (Wild, 1999) was used to assess autonomy support, warmth, and psychological control. The autonomy support subscale had two facets: parental acknowledgment of child’s perspective and encouragement of child-initiated activities. The first facet contained three items (e.g., “My parents often ask me for my opinion”, *ω*_T1_ = 0.66, *ω*_T3_ = 0.77, and *ω*_T4_ = 0.74), and the second facet contained four items (e.g., “My parents encourage me to think about what I want to see on television”, *ω*_T1_ = 0.77, *ω*_T3_ = 0.68, and *ω*_T4_ = 0.69). The perceived parental warmth subscale consisted of four items. An example item is “My parents take care of me when I have problems” (*ω*_T1_ = 0.86, *ω*_T3_ = 0.89, and *ω*_T4_ = 0.88). The perceived psychological control subscale consisted of four items (e.g., “If I don’t do what they tell me right away, something bad happens”; *ω*_T1_ = 0.76, ω_T3_ = 0.76, and *ω*_T4_ = 0.78). All items were rated on a four-point scale (1 = *strongly disagree*, 4 = *strongly agree*).

#### Parental conditional regard

The two facets of PCR––PCNR and PCPR––were measured using four items, each adapted from previous work (Assor et al., 2004; Roth, 2008). Due to the domain-specific nature of these constructs, the content of these items included affective, academic, and behavioral aspects. An example item of PCNR is “My parents would like me less if I didn’t work hard at school”. An example item of PCPR is “My parents would like me more than usual if I was more successful at school”. Responses were rated on a rating scale of 1 = *strongly disagree* to 4 = *strongly agree*. McDonald’s *ω* of both scales was good (for PCNR: *ω*_T1_ = 0.88, *ω*_T3_ = 0.89, and *ω*_T4_ = 0.92; for PCPR: *ω*_T1_ = 0.89, *ω*_T3_ = 0.92, and *ω*_T4_ = 0.91).

#### Academic achievement

Participants were asked to disclose their latest grades on their last report card in math and German. It should be noted that school grades are classified into six levels in Germany, one being the best grade and six the worst. Therefore, lower scores on school grades indicate higher academic achievement. The average grades (i.e., the mean scores of math and German grades) at T3 and T4 were considered outcomes in the data analysis.

#### Psychopathology and prosocial behavior

The self-report and parent report of the Strengths and Difficulties Questionnaire (SDQ; R. Goodman, 1997) were used for assessing internalizing and externalizing problems as well as prosocial behavior at T3 and T4. The German version is accessible at http://www.sdqinfo.org/. This scale contained 25 items that were assigned to Emotional Symptoms (e.g., “Many fears, easily scared”), Conduct Problems (e.g., “Steals from home, school or elsewhere”), Hyperactivity (e.g., “Easily distracted, concentration wanders”), Peer Relationship Problems (“Picked on or bullied by other youths”), and Prosocial Behavior (e.g., “Considerate of other people’s feelings”). The mean score of Emotional Problems and Peer Relationship Problems represented Internalizing Problems, and the mean score of Conduct Problems and Hyperactivity represented Externalizing Problems (A. Goodman et al., 2010). Participants rated each statement on a three-point scale (0 = *strongly disagree*, 1 = *partly agree*, 2 = *strongly agree*). In this study, internal consistency estimates for all subscales varied between *ω* = 0.71 and 0.83 in adolescent reports and parent reports, respectively.

#### Covariates

Adolescents’ gender, school type, migration background, and socioeconomic background were taken into consideration as covariates. Gender was dummy coded as 0 = *girl* and 1 = *boy*. School type was coded as 0 = *Hauptschule* (i.e., the lowest school track) and 1 = *Gymnasium* (i.e., the highest school track, academic track). Migration background was also dummy coded. A student was categorized into 1 (*with migrant background*) if they or at least one of their parents was not born in Germany. In other cases, the student was classified as 0 (*no migrant background*). Socioeconomic status was determined by asking students to rate the number of books in the household on a five-point rating scale ranging from 1 = *0–10 books* to 5 = *over 200 books* (OECD, 2009). Various studies show that this construct is one of the best single indicators of socioeconomic status of the family (Chiu & McBride-Chang, 2006).

### Analysis Strategy

Data analyses were guided by a previous longitudinal person-oriented study (Tang et al., 2021) and an instructional paper (Morin & Litalien, 2017). To address our research questions, we performed longitudinal measurement invariance models, Latent Profile Analysis (LPA) models, and Latent Transition Analysis (LTA) models in *M*plus 8.6 (Muthén & Muthén, 1998–2021).

#### Longitudinal measurement invariance

In the first step, Confirmatory Factor Analysis (CFA) models were run for the six parenting facets (i.e., acknowledgment of the child’s view, encouragement of child-initiated activities, warmth, psychological control, PCNR, and PCPR) using multiple indicators for each measurement time. Once all the cross-sectional CFA models were sound, the next step was to test their longitudinal measurement invariance by stepwise constraining factor loadings, measurement intercepts, and measurement residuals. After the final model of measurement invariance was established, the factor scores (estimated in standardized units as *M* = 0, *SD* = 1) of these parenting facets were saved for further analyses. In contrast to the scale scores, the use of factor scores enables partial control over measurement errors while simultaneously retaining the underlying nature of the measurement model.

#### Latent profile analyses

For Research Objective 1, cross-sectional LPA models for each measurement occasion were carried out. The number of profiles should be determined on the basis of theories and supported by statistical criteria. Statistical evaluation relied on Akaike’s information criterion (AIC), the Consistent AIC (CAIC), the Bayesian information criterion (BIC), the adjusted BIC (ABIC), entropy, and the Lo–Mendell–Rubin likelihood ratio test (LMR-LRT). Models with smaller AIC, CAIC, BIC, and ABIC values should be preferred. Elbow-plots were also used for visualizing the changes in these criteria to assist the decision. A higher entropy value, which ranges from 0 to 1, indicates higher classification accuracy. A significant LMR-LRT suggests that a given *k* profile model significantly improves model fit in comparison to the *k*-1 profile model.

#### Latent transition analyses

After the number of profiles for each wave was determined, these cross-sectional LPA models were integrated into longitudinal LPA models to test profile similarity in four steps: (1) Configural similarity was tested to examine whether the number of profiles was the same over time, based on the same indicators without any constraints; (2) structural similarity was examined by constraining indicator intercepts over time; (3) dispersion similarity was tested by additionally constraining indicator variances over time; and (4) distributional similarity was tested by further constraining profile probabilities over time. For evaluating the models, CAIC, BIC, and ABIC were used. According to these criteria, the model with lower values in two or more of these indices is the most similar model (Morin & Litalien, 2017). After the most similar model was established, it was converted into a longitudinal LTA model to identify the stability and changes across latent profile membership over time (Research Objective 2).

To address Research Objective 3, each of the seven outcomes (i.e., externalizing problems, internalizing problems, prosocial behavior from the adolescent’s perspective, externalizing problems, internalizing problems, prosocial behavior from the parental perspective, and self-reported academic achievement) was added to the LTA model to test *explanatory similarity* (i.e., whether the association between profile membership and outcomes was the same over time). In the LTA models, the seven outcomes at T3 were predicted by parenting profiles at T1, whereas the outcomes at T4 were predicated by parenting profiles at T3. The manual auxiliary three-step approach was used (Asparouhov and Muthén 2014). While estimating the explanatory similarity, the four covariates (i.e., gender, school type, migration background, and socioeconomic background) were controlled. Each of them was incorporated into the final longitudinal LTA model (i.e., *predictive similarity*, whether the effects of covariates on profile membership were stable over time).

### Missing Data Analysis

For missing data analysis, T1 data from participants who remained in all three waves were compared with T1 data from participants who dropped out at either T3 or T4. The original data consisted of 1465 adolescents at T1. From T1 to T3, 390 adolescents dropped out of the project, and an additional 231 adolescents dropped out at T4. A total of 884 adolescents continued for all three waves (418 girls, 218 *Hauptschule* students; 55 of them showed missing values on one or more complete scales of the six parenting dimensions and were excluded from further analysis), whereas 621 adolescents (244 girls, 362 *Hauptschule* students) dropped out at either T3 or T4. Boys were more likely to drop out than girls (*χ*^2^ = 14.96, *p* < 0.001), and *Hauptschule* students were more likely to drop out than *Gymnasium* students (*χ*^2^ = 131.17, *p* < 0.001). Compared to the adolescents who stayed in all three waves, those who dropped out reported significantly lower values in socioeconomic status (*t* = −9.17, *p* < 0.001), lower academic achievement (*t* = 8.28, *p* < 0.001), lower parental warmth (*t* = −3.07, *p* < 0.01), higher psychological control (*t* = 3.77, *p* < 0.001), higher PCNR (*t* = 3.83, *p* < 0.001), and higher PCPR (*t* = 5.42, *p* < 0.001). In terms of perceived parental acknowledgment of the child’s view and encouragement of child-initiated actives, there were no significant differences between the two groups.

A total of 1014 parents of the adolescents also participated in the project at T1. Among these, 579 and 533 parents of selected adolescents continued at T3 and T4, respectively. Parents who dropped out at T3 (*t* = −4.38, *p* < 0.001) or T4 (*t* = −4.83, *p* < 0.001) reported lower levels of education than those who remained for further analyses. In both waves, the parents of the boys and the parents of the *Hauptschule* students were more likely to drop out than the parents of the girls and the parents of the *Gymnasium* students (*χ*^2^ = 8.91 to 129.6, *p*s < 0.01). To handle the missing data, the robust maximum likelihood (MLR) was used throughout the analyses as full-information estimator (Enders, 2010).

## Results

Table 1 presents the zero-order intercorrelations of parenting dimensions and outcomes. Following the outlined analysis strategy, we tested the measurement invariance of the six parenting facets across three waves before performing the LPA and LTA models. Appendix 2 shows these results. The factor scores of these parenting dimensions were used in further analyses.

**Table 1:** Scale means (in parentheses) and zero-order correlations between variables used in present study

	Variable		1	2	3	4	5	6	7	8	9	10	11	12	13	14	15	16
T1	1	AKNO	(3.17)															
	2	INIT	0.40**	(2.95)														
	3	WARM	0.57**	0.33**	(3.48)													
	4	PSY.CONT	−0.23**	−0.14**	−0.34**	(2.33)												
	5	PCNR	−0.16**	−0.09*	−0.19**	0.27**	(1.84)											
	6	PCPR	−0.16**	−0.11**	−0.28**	0.36**	0.56**	(2.11)										
T3	7	AKNO	0.32**	0.15**	0.39**	−0.22**	−0.19**	−0.19**	(3.14)									
	8	INIT	0.17**	0.19**	0.20**	−0.11**	−0.08*	−0.07*	0.41**	(2.77)								
	9	WARM	0.30**	0.14**	0.47**	−0.27**	−0.23**	−0.24**	0.60**	0.36**	(3.40)							
	10	PSY.CONT	−0.16**	−0.09*	−0.23**	0.39**	0.18**	0.23**	−0.29**	−0.12**	−0.34**	(2.13)						
	11	PCNR	−0.10**	−0.13**	−0.20**	0.24**	0.34**	0.38**	−0.23**	−0.11**	−0.32**	0.29**	(1.58)					
	12	PCPR	−0.17**	−0.14**	−0.25**	0.28**	0.32**	0.46**	−0.22**	−0.08*	−0.32**	0.36**	0.61**	(1.81)				
	13	EXTa	−0.19**	−0.06	−0.27**	0.25**	0.13**	0.21**	−0.34**	−0.18**	−0.35**	0.28**	0.13**	0.25**	(0.65)			
	14	INTa	−0.11**	−0.08*	−0.18**	0.20**	0.10**	0.14**	−0.12**	0.0	−0.19**	0.26**	0.12**	0.21**	0.22**	(0.51)		
	15	PROa	0.12**	0.04	0.16**	−0.15**	−0.09**	−0.10**	0.35**	0.29**	0.33**	−0.11**	−0.02	−0.07	−0.39**	−0.02	(1.49)	
	16	EXTp	−0.02	−0.04	−0.18**	0.23**	0.17**	0.21**	−0.23**	−0.10*	−0.24**	0.20**	0.16**	0.22**	0.42**	0.06	−0.16**	(0.38)
	17	INTp	0.05	0.0	−0.12**	0.15**	0.11**	0.14**	−0.02	−0.04	−0.06	0.06	0.1**	0.16**	0.08	0.37**	−0.07	0.09
	18	PROp	0.10*	0.02	0.13**	−0.20**	−0.05	−0.07	0.19**	0.17**	0.22**	−0.11**	−0.13**	−0.13**	−0.23**	0.00	0.30**	−0.23**
	19	ACH	−0.07	−0.07*	−0.13**	0.12**	0.13**	0.21**	−0.15**	−0.15**	−0.15**	0.09*	0.14**	0.22**	0.26**	0.10**	−0.19**	0.38**
T4	21	AKNO	0.23	0.14**	0.23**	−0.18**	−0.10**	−0.08*	0.41**	0.25**	0.35**	−0.20**	−0.13**	−0.11**	−0.16**	−0.12**	0.20**	−0.16**
	22	INIT	0.14**	0.17**	0.15**	−0.07	−0.04	−0.04	0.27**	0.37**	0.22**	−0.06	−0.09**	−0.07	−0.10**	−0.02	0.18**	−0.10**
	23	WARM	0.21**	0.08*	0.32**	−0.20**	−0.13**	−0.17**	0.34**	0.23**	0.48**	−0.21**	−0.22**	−0.20**	−0.22**	−0.16**	0.15**	−0.22**
	24	PSY.CONT	−0.01	−0.06	−0.08*	0.31**	0.19**	0.20**	−0.13**	−0.07*	−0.14**	0.42**	0.20**	0.24**	0.16**	0.18**	−0.04	0.16**
	25	PCNR	−0.12**	−0.14**	−0.20**	0.27**	0.28**	0.33**	−0.16**	−0.10**	−0.24**	0.22**	0.37**	0.43**	0.15**	0.16**	−0.07*	0.15**
	26	PCPR	−0.14**	−0.11**	−0.20**	0.20**	0.28**	0.38**	−0.15**	−0.08*	−0.20**	0.19**	0.34**	0.48**	0.14**	0.19**	−0.08*	0.14**
	27	EXTa	−0.13**	−0.06	−0.15**	0.15**	0.05	0.09*	−0.22**	−0.09*	−0.20**	0.16**	0.06	0.09*	0.57**	0.18**	−0.23**	0.57**
	28	INTa	−0.07*	−0.08*	−0.11**	0.11**	0.05	0.07	−0.06	0.02	−0.11**	0.09*	0.08*	0.13**	0.12**	0.57**	0.05	0.12**
	29	PROa	0.10**	0.03	0.07	−0.10**	−0.07	−0.07	0.26**	0.19**	0.19**	−0.03	−0.03	−0.04	−0.26**	−0.01	0.47**	−0.26**
	30	EXTp	−0.08	−0.06	−0.12**	0.21**	0.06	0.14**	−0.17**	−0.11**	−0.13**	0.19**	0.14**	0.21**	0.43**	0.08	−0.16**	0.43**
	31	INTp	0.00	−0.02	−0.09*	0.10*	0.08	0.14**	−0.08	−0.05	−0.09*	0.05	0.09	0.15**	0.12**	0.29**	−0.02	0.12**
	32	PROp	0.04	0.09*	0.03*	−0.08	0.00	0.00	0.11*	0.19**	0.12**	−0.06	−0.05	−0.09*	−0.16**	0.00	0.28**	−0.16**
	33	ACH	−0.08*	−0.04	−0.12**	0.12**	0.09*	0.16**	−0.17**	−0.12**	−0.13**	0.06	0.07*	0.20*	0.30**	0.04	−0.20**	0.38**

	Variable		17	18	19	21	22	23	24	25	26	27	28	29	30	31	32	33
	17	INTp	(0.28)															
	18	PROp	−0.25**	(1.65)														
	19	ACH	0.18**	−0.10*	(2.71)													
T4	21	AKNO	−0.05	0.09*	−0.12**	(3.15)												
	22	INIT	0.01	0.03	−0.11**	0.44**	(2.73)											
	23	WARM	−0.06	0.12**	−0.11**	0.52**	0.29**	(3.41)										
	24	PSY.CONT	0.07	−0.04	0.03	−0.29**	−0.09*	−0.31**	(2.10)									
	25	PCNR	0.17**	−0.22**	0.10**	−0.25**	−0.08*	−0.34**	0.40**	(1.56)								
	26	PCPR	0.17**	−0.13**	0.17**	−0.25**	−0.07*	−0.38**	0.39**	0.73**	(1.68)							
	27	EXTa	0.01	−0.10*	0.18**	−0.29**	−0.14**	−0.29**	0.20**	0.17**	0.19**	(0.59)						
	28	INTa	0.28**	−0.01	0.05	−0.18**	−0.02	−0.24**	0.19**	0.14**	0.18**	0.13**	(0.52)					
	29	PROa	0.05	0.23**	−0.14**	0.32**	0.26**	0.18**	0.00	−0.04	−0.04	−0.35**	0.01	(1.54)				
	30	EXTp	0.28**	−0.30**	0.32**	−0.11**	−0.05	−0.15**	0.15**	0.20**	0.22**	0.47**	0.10*	0.20**	(0.34)			
	31	INTp	0.65**	−0.20**	0.17**	−0.05	−0.04	−0.06	0.08	0.10*	0.14**	0.08	0.35**	−0.03	0.36**	(0.26)		
	32	PROp	−0.17**	0.56**	−0.07	0.09**	0.08	0.17**	−0.06	−0.21**	−0.16**	−0.19**	−0.04	0.25**	−0.39**	−0.23**	(1.67)	
	33	ACH	0.13**	−0.11**	0.63**	−0.13**	−0.07*	−0.10**	0.01	0.11**	0.17**	0.28**	0.02	−0.18**	0.41**	0.16**	−0.11*	(2.89)

*Note*. AKNO acknowledgment of child’s view, INIT encouragement of child-initiated activities, WARM warmth, PSY.CONT psychological control, PCNR parental conditional negative regard, PCPR parental conditional positive regard, EXTa externalizing problems (adolescent report), INTa internalizing problems (adolescent report), PROa prosocial behavior (adolescent report), EXTp externalizing problems (parent report), INTp internalizing problems (parent report), PROp prosocial behavior (parent report), ACH average school grade

*p < 0.05

**p < 0.01

### Latent Profiles of Parenting Dimensions

**Fig. 1:**
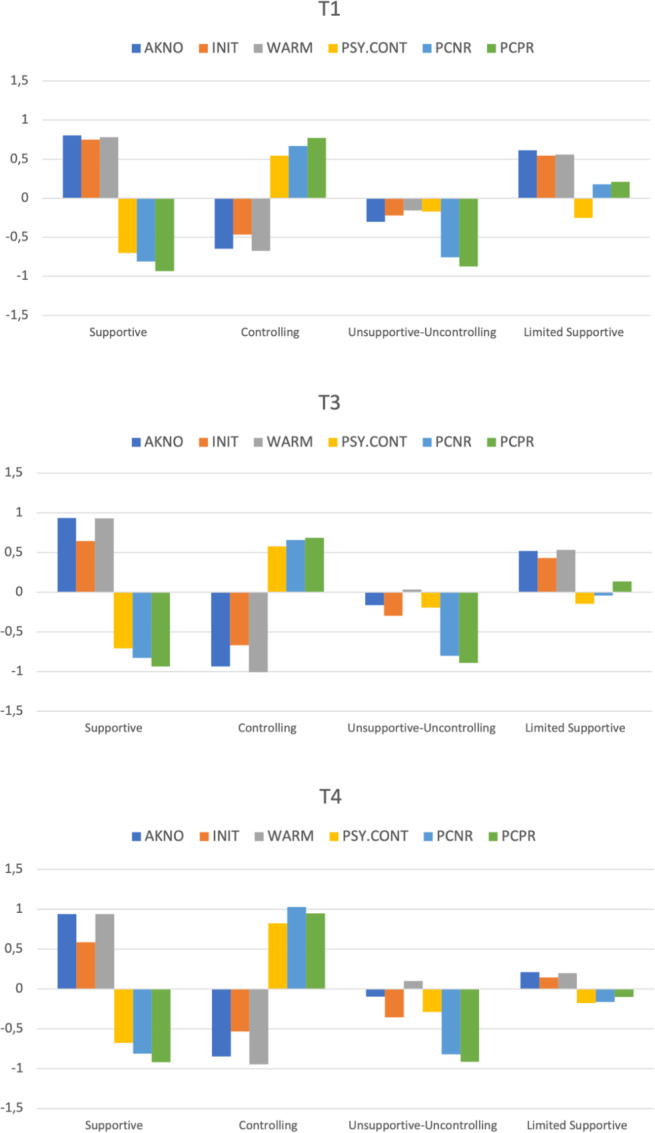
Parenting profiles at T1, T3, and T4 based on partial distributional similarity model. Note. AKNO acknowledgment of child’s view, INIT encouragement of child-initiated activities, WARM warmth, PSY.CONT psychological control, PCNR parental conditional negative regard, PCPR parental conditional positive regard

Following typical practices, solutions with varying numbers of groups were explored, and the one that made the most sense in terms of theory, a priori hypothesis, interpretability, the nature of the profiles, and statistical conformity of the alternative solutions was selected (Marsh et al. 2009). The results of cross-sectional LPA suggested that a four-profile solution was supported by fit indices at T1 and T4 with lower AIC, CAIC, BIC, and ABIC as well as nonsignificant LMR-LRTs if the number of profiles was increased to five (Appendix 3). For T3, fit indices continued to decrease, and the LMR-LRTs remained significant until the number of profiles increased to seven. However, for all three waves, the elbow-plots (Appendix 4) showed that AIC, CAIC, BIC, and ABIC dropped more slowly from a profile number of five. Furthermore, solutions with five or more profiles were not as interpretable as the four-profile solution. A four-profile solution showed high classification accuracy for all three waves (Entropy > 0.89). The proportion of the smallest profiles was around 8%. As a result, a four-profile solution for all three measurement occasions was decided. Next, the similarity of these three cross-sectional LPA models was compared (see Table 2). A partial distributional similarity model was supported (Fig. 1). All further analyses were based on this final partial distributional similarity model.

**Table 2:** Results from final latent profile analysis models and latent transition analysis models

Model	LL	#fp	Scaling	AIC	CAIC	BIC	ABIC	Entropy
*Final Cross-Sectional Latent Profile Analyses*								
T1: 5^th^ grade (4 profiles)^ns^	−4469.287	51	1.2971	9040.575	9329.783	9278.784	9116.832	0.919
T3: 7^th^ grade (4 profiles)	−4127.932	51	1.3443	8357.863	8647.073	8596.073	8434.121	0.905
T4: 9^th^ grade (4 profiles)^ns^	−3885.527	51	1.3471	7873.055	8162.263	8111.264	7949.312	0.894
*Longitudinal Latent Profile Analyses*								
Configural Similarity	−12482.746	153	1.3295	25271.493	26139.119	25986.120	25500.265	0.906
Structural Similarity	−12733.970	105	1.5164	25677.940	26273.370	26168.370	25834.940	0.898
Partial Structural Similarity	−12696.352	111	1.5822	25614.704	26244.159	26133.159	25780.676	0.886
Partial Dispersion Similarity	−12792.742	63	1.7724	25711.484	26068.742	26005.742	25805.684	0.898
Partial Distributional Similarity	−12798.198	59	1.8730	25714.395	26048.971	25989.970	25802.614	0.897
*Latent Transition Analysis*	−2813.584	36	1.0174	5699.169	5903.316	5867.270	5752.952	0.856
*Final Explanatory Similarity Analyses*^*M4*^								
Externalizing problems (S)	−3125.301	42	1.0249	6334.601	6572.454	6530.453	6397.082	0.857
Internalizing problems (S)	−3028.965	42	1.0234	6141.931	6380.049	6337.782	6204.411	0.854
Prosocial behavior (S)	−3415.236	42	1.0178	6914.472	7152.324	7110.324	6976.953	0.856
Externalizing problems (P)	−2842.455	42	1.0295	5768.909	6006.762	5964.761	5831.390	0.854
Internalizing problems (P)	−2823.317	42	1.0639	5730.634	5968.486	5926.486	5793.115	0.854
Prosocial behavior (P)	−3073.152	42	1.0283	6230.303	6468.156	6426.155	6292.784	0.855
Achievement (S)	−4954.033	49	0.9920	10006.067	10229.918	10234.560	10078.961	0.854

*Note*. ^ns^Lo–Mendell–Rubin adjusted LRT Test was not significant. ^*M4*^ In explanatory similarity analysis models, invariant relations with outcome (with covariates) showed the best model fit for all outcomes. S self-report, P parent report, LL model loglikelihood, #fp number of free parameters. Scaling scaling correction factor associated with robust maximum likelihood estimates, AIC Akaike information criteria, CAIC consistent AIC, BIC Bayesian information criteria, ABIC sample size adjusted BIC

Adolescents in Profile 1 (P1) reported high levels of all three supportive parenting facets (i.e., acknowledgment of the child’s view, encouragement of child-initiated activities, and warmth) and low levels of all three controlling facets (psychological control, PCNR, and PCPR). This profile was labeled *Supportive* (12.42% at T1, 17.62% at T3, and 19.77% at T4). In Profile 2 (P2), the three controlling facets were high, but the three supportive facets were low. This profile was labeled *Controlling* (40.56% at T1, 29.91% at T3, and 21.29% at T4). Adolescents in Profile 3 (P3) reported moderately low levels of supportive facets and psychological control and low levels of conditional regard (8.57% at T1, 19.27% at T3, and 21.67% at T4). This profile was named *Unsupportive-Uncontrolling*. In Profile 4 (P4), adolescents reported moderate levels of supportive facets and moderately low levels of psychological control, whereas both forms of conditional regard were moderately high and tended to decrease from T1 to T4. Compared to P1, in which all controlling facets were consistently low, conditional regard occurred more frequently in P4. P4 was named *Limited Supportive* (38.28% at T1, 33.21% at T3, and 37.26% at T4).

### Parenting Profiles’ Stability and Changes

Following the manual auxiliary three-step approach, the final partial distributional similarity model from the last step was converted into an LTA model. Table 3 presents the transition probability matrices for parenting patterns from T1 to T3 and from T3 to T4. The stability of the *Supportive* profile was 50.8% from T1 to T3 and 59.1% from T3 to T4. The probability of its transition to the *Controlling* profile was under 2%, whereas around 20% of the *Supportive* profile became *Unsupportive-Uncontrolling* or *Limited Supportive* over time. Similarly, the *Controlling* profile was comparably relatively stable (stability of 55.5% from T1 to T3 and 50.5% from T3 to T4). This profile rarely changed to *Supportive* across the three waves (under 3%). However, about 40% of the *Controlling* profile changed to either *Unsupportive-Uncontrolling* or *Limited Supportive*. In comparison, the stability of the *Unsupportive-Uncontrolling* and *Limited Supportive* profiles was somewhat lower (stability between 40–51%). From T1 to T3, the probability of shifting from *Unsupportive-Uncontrolling* to each of the other profiles was about 20%. From T3 to T4, the probability of shifting from *Unsupportive-Uncontrolling* to *Limited Supportive* increased to 32.6%, and the probability of shifting from *Unsupportive-Uncontrolling* to *Controlling* decreased to 8%. Compared to the *Unsupportive-Uncontrolling* profile, the *Limited Supportive* profile was slightly more stable. Yet, 14–23% of the *Limited Supportive* profile changed into each of the other profiles across the three waves.

**Table 3:** Transition probabilities (in %) of parenting profiles over time

	1	2	3	4	1	2	3	4
	From T1 to T3				From T3 to T4			
1 Supportive	**50.8**	0.2	25.5	23.8	**59.1**	1.6	18.2	21.1
2 Controlling	2.6	**55.5**	16.8	25.1	0.5	**50.5**	14.9	34.1
3 Unsupportive-Uncontrolling	20.6	18.3	**40.7**	20.4	16.5	8	**43.2**	32.6
4 Limited Supportive	22.5	16.6	14.7	**46.2**	17.2	14.3	17.7	**50.8**

*Note*. Stability estimates are in boldface. Transition probabilities add up to 100% across rows for each measurement point

### Parenting Profiles and Adolescent Development

Explanatory similarity models based on the partial distributional LTA model were performed to address the effects of parenting practices on adolescent development. Appendix 5 provides detailed information on the models with and without constraints. In all the final explanatory similarity models, we controlled for adolescent gender, school type, migration background, and socioeconomic status. The results in Table 4 show that adolescents in the *Controlling* profile reported the highest scores in externalizing and internalizing problems. Adolescents in the *Controlling* and *Unsupportive-Uncontrolling* profiles disclosed the same lowest values in prosocial behavior. Parents of adolescents in the *Unsupportive-Uncontrolling* and *Limited Supportive* profiles reported the same levels of internalizing problems. There were no significant differences between the parent reports on externalizing problems in the *Controlling* and *Unsupportive-Uncontrolling* profiles. Furthermore, adolescents in the *Supportive* profile showed the best academic achievement of all four profiles, and like their parents, they reported the fewest externalizing and internalizing problems and the highest values in prosocial behavior.

**Table 4:** Associations between profile membership and outcomes after controlling for covariate*s*

	Supportive (P1)	Controlling (P2)	Unsupportive-uncontrolling (P3)	Limited supportive (P4)	Summary of significant differences
Outcome	*M* [CI]	*M* [CI]	*M* [CI]	*M* [CI]	*M* [CI]
Externalizing problems (S)	0.447 [0.359; 0.534]	0.762 [0.673; 0.852]	0.624 [0.534; 0.713]	0.560 [0.474; 0.645]	P1 < P4 < P3 < P2
Internalizing problems (S)	0.498 [0.419; 0.577]	0.701 [0.620; 0.781]	0.585 [0.503; 0.666]	0.597 [0.522; 0.672]	P1 < P3 = P4 < P2
Prosocial behavior (S)	1.791 [1.692; 1.890]	1.496 [1.397; 1.596]	1.556 [1.451; 1.660]	1.699 [1.605; 1.793]	P2 = P3 < P4 < P1
Externalizing problems (P)	0.260 [0.175; 0.345]	0.413 [0.322; 0.503]	0.306 [0.215; 0.397]	0.314 [0.231; 0.387]	P1 < P4 < P2; P1 = P3; P3 = P4
Internalizing problems (P)	0.275 [0.185; 0.366]	0.351 [0.270; 0.432]	0.302 [0.215; 0.388]	0.306 [0.224; 0.387]	P1 < P2 = P3 = P4
Prosocial behavior (P)	1.882 [1.743; 2.021]	1.656 [1.507; 1.804]	1.758 [1.608; 1.908]	1.749 [1.611; 1.886]	P3 = P4 < P2 < P1
Academic achievement (S)^a^	2.993 [2.820; 3.166]	3.187 [3.016; 3.357]	3.159 [2.979; 3.339]	3.103 [2.936; 3.270]	P1 < P4 < P3 = P2

*Note*. *M* mean value, *CI* 95% confidence interval of mean value, *S* self-report, *P* parent report

^a^It should be noted that lower school grades indicate higher academic achievement in Germany

## Discussion

Although parenting profiles have been the focus of research for several decades, little is known about their stability and changes or their longitudinal effects on development. By incorporating four autonomy-related parenting dimensions (i.e., autonomy support, warmth, psychological control, and conditional regard) from Self-Determination Theory and using a longitudinal person-oriented approach, this study contributes substantially to the understanding of autonomy-related parenting profiles and their stability and effects. As expected, four parenting profiles were identified. Adolescents whose parents provided a fully autonomy-supportive climate reported the best academic achievement, the lowest risk of psychopathology, and the highest scores in prosocial behaviors, whereas adolescents whose parents provided a highly controlling climate reported the lowest achievement and the highest risk of psychopathology. Further, parenting profiles were less stable than suggested by the literature.

### Parenting Profiles and their Relationships with Adolescent Development

This study extends the research on autonomy-related parenting profiles by considering the core parenting dimensions from a Self-Determination Theory perspective (i.e., autonomy support, warmth, psychological control, and conditional regard). In total, four parenting profiles were identified, namely *Supportive*, *Controlling*, *Unsupportive-Uncontrolling*, and *Limited Supportive* across three measurement time.

In this study, *Supportive* parents were authentically supportive and consistent in their parenting behaviors. They respected their children’s perspectives, encouraged their children to experiment, were emotionally warm, and used minimal controlling strategies. *Controlling* parents used psychological methods to control their children’s emotions and behaviors and neglected their children’s developmental needs. *Unsupportive-Uncontrolling* parents were emotionally distant from their children, did not take their children’s perspective and needs into consideration, but did not use autonomy-thwarting strategies. These parents seemed to care less about their children’s development in general. Finally, the *Limited Supportive* profile was characterized by moderate autonomy support and warmth, but moderately low psychological control and moderate conditional regard. This result supports the assumption that conditional regard is domain specific, and that parents can be generally supportive but still use conditional regard (Assor et al., 2014).

Further results showed that, in line with assumptions of Self-Determination Theory (Deci & Ryan, 2000) and previous findings, adolescents who were categorized into the *Supportive* profile reported the fewest externalizing and internalizing problems, the most prosocial behaviors, and the best academic performance. This was the reverse case for adolescents in the *Controlling* profile. Moreover, the adolescents in the *Unsupportive-Uncontrolling* and *Limited Supportive* profiles showed similarly high levels of internalizing problems, and the adolescents in the *Unsupportive-Uncontrolling* profile even reported the same values in prosocial behavior as the adolescents in the *Controlling* profile. Overall, these findings underline the fostering effect of autonomy-supportive parenting characterized by acknowledgment of the child’s perspective, encouragement of child-initiated activities, and warmth; and the harmful effect of controlling parenting characterized by psychological control, and positive and negative conditional regard. Our results further highlight that low control cannot compensate for the detrimental effects of low autonomy support. Moreover, it was possible to generalize these findings to conditional regard as a specific form of parental control that many parents and some previous researchers see as beneficial (e.g., Frost, 2005; McGraw, 2005; Steinberg, 2004). Using positive and negative conditional regard despite being autonomy-supportive is among the most maladaptive styles in terms of internalizing problems.

Moreover, the findings concerning the *Limited Supportive* parenting profile indicate that parental autonomy support and warmth should be granted unconditionally. In other words, parental regard should not be contingent on the child’s fulfillment of parental demands and expectations in specific domains. Further, parental support should be at a high level to optimally promote adolescents’ academic and psychological adjustment. A possible explanation is that a secure and trustworthy parent–child relationship can only be established when parents provide full support to their adolescents (Ryan et al., 2006). This then allows adolescents the freedom to explore their interests, identity, and world without the concerns of punishment, withdrawal of love, or failure (Assor et al., 2014). Limited support of autonomy unfortunately does not reduce adolescents’ concerns and has little effect on autonomy building.

It is important to note that these findings were retrieved from two sets of informants––adolescents and their parents. The bivariate correlations between self- and parent-reported internalizing and externalizing problems as well as prosocial behavior were between 0.25 and 0.47. These correlations were comparable with meta-analytical findings among Western adolescent–parent dyads (De Los Reyes & Kazdin, 2005). Moreover, the results of the parents of the adolescents in the *Supportive* and *Controlling* profile were consistent with their children’s estimates overall, whereas the results concerning significant differences showed that the relationship pattern of adolescents in the *Unsupportive–Uncontrolling* and *Limited Supportive* profiles was slightly different to that of their parents.

### Parenting Profiles’ Stability and Changes during Early and Mid-adolescence

Previous studies have reported that parenting profiles are highly stable (e.g., stability 55–81%; Zhang et al., 2017). The present findings indicate that autonomy-related parenting profiles are less stable than these previous findings suggest. In this study, only around half of the *Supportive* and *Controlling* profiles remained stable from early adolescence to mid-adolescence, whereas the stability of the *Unsupportive–Uncontrolling* and *Limited Supportive* profiles was even lower. Yet, it should be positively noted that supportive parenting became more stable over time during mid-adolescence, whereas more parents who were controlling during their child’s early adolescence became less controlling during mid-adolescence. In Germany, the transition from elementary to secondary school takes place after the completion of the fourth grade (age: ca. 10 years). In the present study, T1 was conducted during the second term of the fifth grade, when students were still new in their new schools. The associated academic and environmental changes can be a potential stressor and may affect parenting behaviors and students’ receptiveness to these behaviors. Moreover, previous scholars have shown that in different stages of adolescence, changes vary in quantity and quality across different domains. For example, compared to mid-adolescence, early adolescence is associated with more unstable parent-adolescent relationships (e.g., more relationship conflicts) and lower adaptive emotion regulation competence (e.g., Zimmermann & Iwanski, 2014). The findings of the present study are also compatible with the transactional model of parent–child interaction (Kuczynski & Parkin, 2007; Sameroff, 2010), and it can be speculated that the extent of the changes in parenting profiles may be dependent upon the changes in their adolescent children’s emotions, motivations, and personalities. With adolescents’ increasing stability of emotions and more realistic self-estimation, parents may have more trust in their adolescent children, provide more autonomy, and become less controlling. For parents, adolescence is also a journey of discovery in which they explore their parental role and adapt their parenting. In addition to adolescents’ characteristics, parenting behaviors are affected by a variety of factors, such as parents’ beliefs, experience, and contextual conditions (Epstein & Sanders, 2002; Hoover-Dempsey & Sandler, 1997, 2005). Supported by the current findings, assuming that parenting is a stable trait is problematic. Future studies should pay more attention to the mechanism of parenting profiles’ stability and changes.

### Practical Implications

As the first longitudinal study on autonomy-related parenting profiles and their effects on German adolescents in the period from early to mid-adolescence to include both adolescent’s self-reports and parent reports, these findings have several important practical implications. First, although it is an encouraging finding that parents appear to become more autonomy-supportive and less controlling during adolescent development, almost two-thirds of parents were highly controlling or gave limited support in their parenting across all the three measurement points from the perspective of adolescents. They used psychological control and/or showed conditional regard. The proportion of *Supportive* parenting profile was relatively small (i.e., 12–20% across the three measurement points). Growing up in a controlling environment, adolescents tend to show poor academic and psychological adjustment and are at a high risk of internalizing and externalizing problems. From the perspective of clinical and developmental psychology, adolescence is seen as a critical stage of the manifestation of psychopathology (e.g., Klipker et al., 2018). In Germany, the prevalence of any psychological disorder among young people is about 20%, and those from low socioeconomic families are particularly vulnerable (Klipker et al., 2018). Therefore, prevention and intervention programs for adolescents that help them manage educational transitions, aid their emotion regulation, and promote their psychological adjustment could be beneficial. The German education system is characterized by a high degree of social inequality (OECD, 2019). In the case of students from low socioeconomic families, schools and communities are encouraged to provide students and their parents with more social support and assessable consulting services.

Another encouraging finding was that it is possible for parents to adopt more autonomy-supportive parenting strategies and that it is not too late to provide parents with guidance on positive parenting skills through parenting programs. The person-oriented results also indicated that it is important that parenting programs not only focus on adaptive parenting strategies, but also address the various detrimental effects of different forms of dysfunctional strategies. For instance, it would be helpful if parents were informed and aware of the harmful effects of conditional regard, which many parents have seen and some researchers and practitioners have recommended as a positive parenting strategy (e.g., Frost, 2005; McGraw, 2005; Steinberg, 2004).

Third, parents are encouraged to give unconditional and high autonomy support to their offspring and give them complete freedom to explore their “true selves” and make their own experiences. More importantly, parents are advised to avoid providing a moderate level of autonomy support. Providing a limited or moderate level of autonomy support is unfortunately as maladaptive as providing non-autonomy support.

### Limitations and Future Research

Several limitations need to be taken into consideration. First, the scales used for measuring PCNR and PCPR included affective, academic, and behavior domains. As Assor et al. (2014) suggested, some parents may practice contingent regard according to more fine-grained domains. For example, parents may show attention and love when their child receives good school assessment results, but their attention does not depend upon whether their child is good at sports or vice versa. In the future, it may be worthwhile more differentially testing conditional regard in different domains. Second, although this study included parent reports on the outcomes, we only considered the perception of parenting practices from the perspective of adolescents. It is strongly recommended that future research examines parenting practices from the parents’ perspective. This would allow researchers to examine and control the potential bias in the perception of parenting. It is also feasible to examine the reciprocal relationship between parenting practices and adolescents’ academic and psychological outcomes. Third, to protect adolescents’ privacy, we used self-reported academic achievements instead of a more objective measure. The participants may have intentionally over-inflated their grades, and the retrospective report may have led to inaccuracy. Finally, this study focused on the period from early adolescence to mid-adolescence. It may be interesting to extend the measurement period to cover the whole period of adolescence, to test whether the assumption still holds that parents become more autonomy-supportive as adolescent emotions and personality become more stable.

## Conclusion

There is a consensus that supporting adolescents’ needs for autonomy is crucial to their positive development. However, autonomy-related parenting profiles are largely underrepresented in the long tradition of parenting research. Even less known is about whether and to what extent parenting profiles change. The present study advanced our understanding of parenting by exploring autonomy-related parenting profiles, these profiles’ longitudinal stability and changes, and their effects on adolescents’ academic and psychological development. Four autonomy-related parenting profiles were identified in a German adolescent-parent sample: *Supportive*, *Controlling*, *Unsupporitve*-*Uncontrolling*, and *Limited Supportive*. It can be concluded that being controlling or providing limited or absent autonomy support undermines adolescents’ psychological needs and ultimately increases the risk of psychopathology and impairs academic success. In contrast, supportive parenting, through encouraging adolescents’ self-initiating activities, acknowledging their view, providing emotional support, and using minimal controlling strategies, contributes to adolescents’ positive academic and psychological development. Furthermore, the present findings suggest that parenting profiles are less stable than assumed in previous studies. Overall, this study provides important empirical and practical implications for encouraging supportive parenting. Nevertheless, more longitudinal person-oriented studies are needed in future research.

## Appendix 1. Detailed Information of the Original Sample and the Sample Used in the Present Study

**Table:**

Subsample	Sample size	T1	T3	T4
*Adolescents*				
Original	Total	1465	1378	1441
	Girls	662	624	663
	*Gymnasium* students	885	858	803
	Immigrant students	579	531	594
In this study	Total	789	789	789
	Girls	395	395	395
	*Gymnasium* students	584	584	584
	Immigrant students	245	245	245
*Adolescent-parent dyads*				
Original	Total	1014	871	769
	Girls	455	415	397
	*Gymnasium* students	703	615	538
	Immigrant families	296	249	221
In this study	Total	–	579	533
	Girls	–	291	271
	*Gymnasium* students	–	452	425
	*Mothers*		427	411
	Immigrant families	–	138	137

Note. *T1* 5^th^ grade, in Spring 2010. *T3* 7th grade, in Spring 2012. *T4* 9th grade, in Spring 2014

## Appendix 2. Results from Confirmatory Factor Analysis Models and Their Longitudinal Measurement Invariance

**Table:**

Model	*χ* ^2^	*df*	*p*	CFI	RMSEA	90% CI for RMSEA	SRMR
*Cross-sectional confirmatory factor analysis analyses*							
T1: 5^th^ grade	564.925	215	<0.001	0.929	0.045	0.041/0.050	0.044
T3: 7^th^ grade	507.657	215	<0.001	0.949	0.042	0.037/0.046	0.037
T4: 9^th^ grade	633.055	215	<0.001	0.936	0.0.050	0.045/0.054	0.043
*Longitudinal measurement invariance*							
Configural MI	3478.376	2055	<0.001	0.933	0.030	0.028/0.031	0.039
Weak MI	3649.920	2101	<0.001	0.927	0.031	0.029/0.032	0.050
**Partial strong MI**	3904.132	2128	<0.001	0.917	0.033	0.031/0.034	0.053
Strong MI	4279.501	2147	<0.001	0.900	0.035	0.034/0.037	0.058

Note. *N* 789

The final model is in boldface

## Appendix 3. Results from Latent Profile Analysis Models Estimated Separately at Each Measurement Occasion

**Table:**

	#profiles	LL	#fp	Scaling	AIC	CAIC	BIC	ABIC	Entropy	LMR-LTR
T1: 5^th^ grade	1	−6190.052	12	1.1136	12404.103	12472.153	12460.152	12422.046	N/A	
	2	−5292.387	25	1.7083	10634.774	10776.543	10751.543	10672.155	0.889	0.045
	3	−4795.169	38	1.6892	9666.339	9881.827	9843.828	9723.158	0.901	0.012
	**4**	−4469.287	51	1.2971	9040.575	9329.783	9278.784	9116.832	0.919	0.012
	5	−4256.795	64	1.5628	8641.589	9004.519	8940.518	8737.285	0.922	0.410
	6	−4095.962	77	1.3101	8345.924	8782.573	8705.573	8461.058	0.905	0.012
	7	−3981.074	90	1.2536	8142.148	8652.517	8562.517	8276.720	0.895	0.163
T3: 7^th^ grade	1	−6264.295	12	1.0988	12552.589	12620.639	12608.638	12570.532	N/A	
	2	−4879.568	25	1.4602	9809.137	9950.905	9925.906	9846.518	0.961	0.000
	3	−4422.782	38	1.3240	8921.564	9137.053	9099.053	8978.383	0.907	0.040
	**4**	−4127.932	51	1.3443	8357.863	8647.073	8596.073	8434.121	0.905	0.004
	5	−3934.059	64	1.3448	7996.119	8359.047	8295.048	8091.815	0.898	0.036
	6	−3785.641	77	1.2221	7725.282	8161.931	8084.931	7840.415	0.912	0.002
	7	−3641.266	90	1.3114	7462.531	7972.901	7882.900	7597.103	0.905	0.098
T4: 9^th^ grade	1	−6138.414	12	1.1472	12300.828	12368.877	12356.877	12318.771	N/A	
	2	−4529.918	25	1.1545	9109.837	9251.605	9226.606	9147.218	0.969	0.000
	3	−4140.347	38	1.4877	8356.693	8572.183	8534.182	8413.512	0.887	0.040
	**4**	−3885.527	51	1.3471	7873.055	8162.263	8111.264	7949.312	0.894	0.003
	5	−3689.991	64	1.4471	7507.983	7870.911	7806.912	7806.912	0.899	0.120
	6	−3558.511	77	1.5839	7271.022	7707.671	7630.671	7386.155	0.882	0.593
	7	−3452.284	90	1.2327	7084.568	7594.937	7504.937	7219.140	0.892	0.208

Note. #profiles number of profiles, *LL* model loglikelihood, #fp number of free parameters, Scaling scaling correction factor associated with robust maximum likelihood estimates, *AIC* Akaike information criteria, *CAIC* consistent AIC, *BIC* Bayesian information criteria, *ABIC* sample size adjusted BIC, *LMR-LTR* Lo–Mendell–Rubin adjusted LRT Test

The selected profile solutions are in boldface

## Appendix 4. Elbow Plot of the Value of the Information Criteria for Solutions including Different Number of Latent Profiles for Each Measurement Occasion

Note. T1 5th grade. T3 7th grade. T4 9th grade. AIC Akaike information criteria. CAIC consistent AIC. BIC Bayesian information criteria. ABIC sample size adjusted BIC

## Appendix 5. Results from Explanatory Similarity in Latent Transition Analysis Models

**Table:**

Outcome	Model	LL	#fp	Scaling	AIC	CAIC	BIC	ABIC	Entropy
Externalizing problems (S)	M1	−3266.799	37	1.0044	6607.597	6817.134	6780.369	6662.875	0.858
	M2	−3271.166	33	1.0365	6608.331	6795.215	6762.425	6657.632	0.858
	M3	−3120.685	46	1.0209	6333.370	6593.874	6547.874	6401.801	0.857
	**M4**	−3125.301	42	1.0249	6334.601	6572.454	6530.453	6397.082	0.857
Internalizing problems (S)	M1	−3202.642	37	1.0129	6479.284	6689.055	6652.055	6534.561	0.855
	M2	−3204.774	33	1.0436	6475.548	6662.641	6629.641	6524.849	0.855
	M3	−3028.650	46	1.0229	6149.299	6410.097	6363.803	6217.730	0.855
	**M4**	−3028.965	42	1.0234	6141.931	6380.049	6337.782	6204.411	0.854
Prosocial behavior (S)	M1	−3539.489	37	0.9911	7152.978	7362.514	7325.749	7208.255	0.857
	M2	−3545.371	33	1.0107	7156.742	7343.625	7310.835	7206.043	0.857
	M3	−3410.552	46	1.0183	6913.105	7173.608	7127.609	6981.536	0.856
	**M4**	−3415.236	42	1.0178	6914.472	7152.324	7110.324	6976.953	0.856
Externalizing problems (P)	M1	−2997.710	37	1.0285	6069.420	6278.956	6242.191	6124.697	0.855
	M2	−2999.422	33	1.0593	6064.845	6251.727	6218.938	6114.146	0.855
	M3	−2841.306	46	1.0357	5774.612	6035.116	5989.116	5843.043	0.854
	**M4**	−2842.455	42	1.0295	5768.909	6006.762	5964.761	5831.390	0.854
Internalizing problems (P)	M1	−2963.942	37	1.0515	6001.883	6211.42	6174.654	6057.160	0.855
	M2	−2966.737	33	1.0865	5999.473	6186.357	6153.567	6048.775	0.854
	M3	−2820.613	46	1.0620	5733.226	5993.73	5947.730	5801.657	0.854
	**M4**	−2823.317	42	1.0639	5730.634	5968.486	5926.486	5793.115	0.854
Prosocial behavior (P)	M1	−3187.298	37	1.0231	6448.596	6658.132	6621.368	6503.873	0.866
	M2	−3188.256	33	1.0501	6442.511	6629.395	6596.605	6491.813	0.855
	M3	−3071.482	46	1.0308	6234.964	6495.468	6449.468	6303.395	0.855
	**M4**	−3073.152	42	1.0283	6230.303	6468.156	6426.155	6292.784	0.855
Achievement (S)	M1	−5377.741	42	0.9830	10839.481	11039.018	11035.600	10902.228	0.855
	M2	−5451.447	34	1.0289	10970.895	11155.777	11129.657	11021.690	0.854
	M3	−4945.892	57	1.0043	10005.784	10244.288	10271.582	10090.579	0.854
	**M4**	−4954.033	49	0.9920	10006.067	10229.918	10234.560	10078.961	0.854

Note. *LL* model loglikelihood, #fp number of free parameters, Scaling scaling correction factor associated with robust maximum likelihood estimates. *AIC* Akaike information criteria, *CAIC* consistent AIC, *BIC* Bayesian information criteria, *ABIC* sample size adjusted BIC. M1 free relations with outcome (without covariates), M2 invariant relations with outcome (without covariates), M3 free relations with outcome (with covariates), M4 invariant relations with outcome (with covariates)

The final models are in boldface
